# A Scale for the Assessment of Sexual Standards Among Youth: Psychometric Properties

**DOI:** 10.1007/s10508-017-1001-x

**Published:** 2017-05-30

**Authors:** Peggy M. J. Emmerink, Regina J. J. M. van den Eijnden, Tom F. M. ter Bogt, Ine Vanwesenbeeck

**Affiliations:** 1 Department of Interdisciplinary Social Sciences, Utrecht University, 3584 CS Utrecht, The Netherlands; 2 Rutgers, Expert Centre for Sexual and Reproductive Health and Rights, Utrecht, The Netherlands

**Keywords:** Adolescents, Sex role attitudes, Sexual double standards, Sexuality, Social norms

## Abstract

The (hetero)sexual double standard (SDS), prescribing sexual modesty for girls and sexual prowess for boys, negatively affects sexual and mental health. Nevertheless, endorsement and enactment of the SDS is still common. In this study, we respond to recent calls for modernization in the field of sexual double standard research. We describe the development of the “Scale for the Assessment of Sexual Standards among Youth” (SASSY), as well as its psychometric properties. This instrument was designed to measure contemporary sexual double standard endorsement, defined as “the degree to which an individual’s attitude reflects a divergent set of expectations for boys and girls, in that boys are expected to be relatively more sexually active, assertive, and knowledgeable and girls are expected to be relatively more sexually reserved, passive, and inexperienced” among adolescents and emerging adults. In Study 1, a pool of 35 items was administered in a Dutch sample (*N* = 465, 54.8% female, age 16–20). A 20-item set formed a one-dimensional and internally consistent scale and was subsequently administered in a second Dutch sample. Study 2 (*N* = 818, 58.4% female, age 16–25) again assessed the 20-item set. After dropping one item, the 19-item SASSY proved to be one-dimensional and internally consistent, exhibiting good test–retest reliability, construct validity, and convergent validity. Finally, the instrument showed configural and metric measurement invariance across gender, age, education level, and sexual experience level, and configural, metric, and scalar measurement invariance across time. These studies confirmed the 19-item SASSY to be a reliable and valid new tool for the assessment of contemporary sexual double standard endorsement among adolescents and emerging adults.

## Introduction

Reiss (1967) conducted the first large-scale study of attitudes toward “various degrees of sexual permissiveness embodied in our premarital standards” (p. 6), in which he noted that egalitarianism had not yet been achieved and that a sexual double standard (SDS) existed for men and women (Crawford & Popp, 2003). Many studies on the sexual double standard followed and the concept has been thoroughly reviewed over recent decades (Bordini & Sperb, 2013; Crawford & Popp, 2003; Eaton & Rose, 2011; Fugère, Escoto, Cousins, Riggs, & Haerich, 2008; Sanchez, Fetterolf, & Rudman, 2012). These reviews conclude that heterosexual romantic relationships seem to have become somewhat more egalitarian, but that the sexual double standard still exists, albeit in a different form. Whereas originally the central notion of the sexual double standard pertained to premarital courting and sexual behavior (Reiss, 1967), later definitions focused less on marital status, and included expectations in terms of sexual roles in line with the sexual double standard (e.g., prescribing divergent [re]active and [sub]assertive sexual roles to men and women) (Sanchez et al., 2012).

The sexual double standard has been related to a multitude of negative sexual and health outcomes, such as increased dating violence and sexual violence (Shen, Chiu, & Gao, 2012), poor sexual functioning among young women (Kiefer & Sanchez, 2007), higher STI/HIV infection risk (Bermúdez, Castro, Gude, & Buela-Casal, 2010), and decreased sexual and relationship satisfaction for both men and women (Sanchez, Crocker, & Boike, 2005). However, other studies in this field have produced mixed and contradictory results (Fugère et al., 2008; Marks & Fraley, 2005, 2006; Sanchez et al., 2012). Reviews have partially ascribed this to methodological issues (Sanchez et al., 2012), as well as to the use of outdated measures (Bordini & Sperb, 2013; Crawford & Popp, 2003; Fugère et al., 2008). It seems that the concept of the sexual double standard has evolved along with changes in the display of gendered behavior in dating and sexuality, but research methods have not been able to keep pace. This calls for the development of modernized methods and measures.

In this study, we respond to this call for modernization (Bordini & Sperb, 2013), by introducing a new measure to examine sexual double standard endorsement in its contemporary form. The new measure was designed based on a number of desired features. Firstly, we set out to develop an instrument that was suitable for capturing sexual double standard endorsement from the moment people begin to experience romantic and partnered sexual situations. Adolescence is a period when people start to explore sexual and romantic interactions (Collins, Welsh, & Furman, 2009), whereas emerging adulthood is a time when romantic interactions become more serious and are more likely, compared to adolescence, to include sexual intercourse (Arnett, 2000). We also know that there is already evidence of sexual double standards in first-time sexual interactions (Sanchez et al., 2012). Simultaneously, older measures mostly asked about abstinence before marriage, which does not translate well to today’s reality for young people as marriage rates fall and different relationship forms, such as co-habitation, are on the rise. Therefore, the suitability of the new instrument specifically for assessment among both adolescents and emerging adults was a key factor in its design.

Secondly, the instrument encompassed a greater variety of aspects of sexual double standards in comparison with older sexual double standard measures. Many previous measures have focused heavily on permissiveness and sexual abstinence before marriage, such as the Sexual Double Standard Scale (Muehlenhard & Quackenbush, 1998) and the Double Standard Scale (Caron, Davis, Halteman, & Stickle, 1993). However, in a contemporary context, the sexual double standard encompasses several other aspects that have been insufficiently highlighted or were absent in previous measures. Based on numerous studies, sexual double standard endorsement is no longer related only to premarital sex and virginity status, but also to (as many as 13) other beliefs relevant to the SDS construct (Allen, 2003; Bay-Cheng, 2015; Hayes, Lorenz, & Bell, 2013; Horne & Zimmer-Gembeck, 2005, 2006; Hyde, 2005; Kehily, 2001; Kreager & Staff, 2009; Moss-Racusin, Phelan, & Rudman, 2010; Petersen & Hyde, 2010; Reidy, Shirk, Sloan, & Zeichner, 2009; Sanchez, Fetterolf, & Rudman, 2012; Sprecher, Regan, & McKinney, 1998; ter Bogt, Engels, Bogers, & Kloosterman, 2010; Tolman, 2009, 2012; Vanwesenbeeck, 2009; Yasan, Essizoglu, & Yildirim, 2009).

We therefore chose to reflect this multifaceted nature of the contemporary sexual double standard in the item pool of the new instrument (Study 1). In doing so, we broadly define the contemporary sexual double standard as “the degree to which an individual’s attitude reflects a divergent set of expectations for boys and girls, in that boys are expected to be relatively more sexually active, assertive, and knowledgeable and girls are expected to be relatively more sexually reserved, passive, and inexperienced” (Emmerink, van den Eijnden, Ter Bogt, & Vanwesenbeeck, 2016). The instrument thus assesses an individual’s attitude toward perceived social norms concerning sexuality for boys and girls. We wish to be mindful of deleting any of the themes established above entirely, through the deletion of items based on statistical arguments, although this cannot be completely prevented. We believe that the leading argument in this case should be that the multifaceted nature of the scale should not be compromised.

Thirdly, since the sexual double standard is a highly heteronormative phenomenon, the instrument was designed specifically for assessment in heterosexual samples. Although non-heterosexual populations are also bound to be affected by heteronormative gender norms (Szymanski & Henrichs-Beck, 2014), it is plausible that they are affected in a different way. It may be possible in the future to adapt the instrument for use in non-heterosexual samples. Fourthly, to enhance comparability of the results for young men and women, as well as to enable the instrument to be used in multiple study types, we designed the instrument so that it would be suitable for assessing both females and males. Fifthly and lastly, the original item pool was constructed in a manner matching our expectation that this instrument would measure a single construct, namely the sexual double standard. The study should show, however, whether this is indeed the case.

This article covers both instrument development (Study 1) and tests of psychometric properties (Study 1 and 2). The following research questions are addressed in the two studies:

1. Which subset of items for assessing sexual double standard endorsement among male and female adolescents and emerging adults forms the best one-factor and internally consistent scale? (Study 1).
2. Does the factor structure of the newly developed instrument established in the first study replicate in another sample, and what are the test–retest reliability, the construct validity, and convergent validity of this instrument? (Study 2).
3. Does the newly developed instrument show measurement (in)variance across time, gender, age, education level, and sexual experience level? (Study 2).

## Study 1

## Method

### Participants

The sample consisted of 512 adolescents and emerging adults (46.9% boys, 53.1% girls), aged between 16 and 20 years (*M* = 18.12, SD = 1.37). Participants completed a survey on “adolescent sexuality” and were assured of anonymity and the ability to end their participation at any time. Based on a sexual orientation question with a five-point response scale, participants were excluded if they indicated that they were attracted exclusively or mainly to members of their own sex, were attracted to both sexes equally, or were undecided as to their sexual orientation. Using this criterion, 47 participants were excluded from the analyses. The final sample consisted of 465 heterosexual adolescents (45.2% boys, 54.8% girls) (which amounts to 90.8% of the original sample), aged between 16 and 20 years (*M* = 18.08, SD = 1.34). Sample characteristics are shown in Table 1.

**Table 1:** Sample characteristics

		Male (*n* = 210)		Female (*n* = 255)	
Age in years (*M*, SD)		18.19 (1.35)		17.98 (1.33)	
Ethnicity (*n*, %)	Native Dutch	159 (75.7%)		178 (69.8%)	
	Of other ethnicity	51 (24.3%)		77 (30.2%)	
Education (*n*, %)	Lower	26 (12.4%)		25 (9.8%)	
	Intermediate	112 (53.3%)		137 (53.7%)	
	Higher	72 (34.3%)		93 (36.5%)	
Sexual experience (*n*, %)	No Yes	93 (44.3%) 117 (55.7%)		88 (34.5%) 167 (65.5%)	

^a^Two scores on religion were missing; valid score percentages are presented

### Procedure

Ethical approval for the study was granted by the Child and Adolescent Studies department board at Utrecht University. An online panel enrolled by a commercial party was contracted to recruit community participants for our study. Participants were able to win prizes by participating, but received no financial reward. The aim was to obtain a sample that included often underrepresented groups (e.g., non-native Dutch and lower educated participants) in order to adequately reflect Dutch society. The method of data collection provided us with a sample of community adolescents and emerging adults aged between 16 and 20 years. The use of a panel made acquiring this sample more feasible. Moreover, using an Internet panel was of added value because our study involved rather personal questions and the Internet offers relative anonymity. This allowed participants to complete the questionnaire in the comfort and privacy of their own homes. Participants ticked a box stating that they understood that the questions would be of a sexual nature and that they wanted to continue to the questionnaire. They were further informed that they could cease their participation at any time. No parental consent was needed, because the minimum age for completing the questionnaire was 16.

### Measures

The proposed scale items were designed with older sexual double standard measures in mind (e.g., Traditional Sexual Attitudes [Kiefer & Sanchez, 2007]; Gender-Equitable Men Scale [Pulerwitz & Barker, 2008]; Male Role Attitudes Scale [Pleck, Sonenstein, & Ku, 1994]; Double Standard Scale, [Caron et al., 1993]; Sexual Double Standard Scale [Muehlenhard & Quackenbush, 1998]) as well as based on empirically and theoretically derived insights from the literature analysis described in the Introduction. We made sure to design items that would be suitable for assessment among heterosexual male and female adolescents and emerging adults (i.e., no difficult wording or too many items describing marriage). In total, we generated 35 items on which participants indicated their degree of agreement on a six-point Likert scale ranging from “1 = completely disagree” to “6 = completely agree.” The items consisted of statements reflecting perceived social norms concerning sexuality for boys and girls. The study was conducted in the Dutch language. To facilitate readability for an international audience, English translations of the items are given in “Original 35-Item Pool for the SASSY”section. The original Dutch item wording can be obtained from the corresponding author upon request.

#### Demographics

*Gender and age* Participants indicated their biological sex (male or female) and age.

*Education* Participants answered a question on their current occupation: studying or not studying. They also indicated the highest academic qualification they had attained. If participants’ main occupation was studying, the type of education they were following was taken as their education level. If participants indicated that they were currently not studying, the highest-level qualification they had obtained was taken as their education level. Education level was categorized as lower (primary school and junior vocational training), intermediate (intermediate education and vocational training), and higher education (pre-university education and university).

*Sexual experience* Participants answered the question, “How many people have you had sex with in your life?” on a five-point scale with response categories ranging from “1 = none” to “5 = more than 10.” A definition of sex was given: “By “sex,” we mean everything from feeling each other naked or caressing each other, to intercourse (penetration of the vagina or anus by the penis).” The responses were then recoded into a binary variable for use in the analyses: no sexual experience (for participants who answered “none”) versus sexual experience (for participants who answered “one or more”).

### Analytical Strategy

We assessed the factor structure and internal consistency of the 35-item pool to determine which subset of items formed the best one-factor and internally consistent, reliable scale for the assessment of sexual double standard endorsement. We employed an exploratory factor analysis to this purpose. There were no missing values; therefore, no missing data handling procedure was needed.

## Results

### Factor Structure and Internal Consistency

First, the factor structure and reliability of the 35-item instrument we had constructed were assessed. The scale was subjected to an exploratory factor analysis using principal axis factoring with oblique rotation. The Kaiser–Meyer–Oklin value was .88, which is above the recommended cutoff value of .60 (Kaiser, 1970, 1974), and Bartlett’s Test of Sphericity (Bartlett, 1954) was statistically significant, supporting factorability. Furthermore, upon inspection of the scree plot, a break could be seen after the first component extracted. As the aim was to construct a one-dimensional (single factor) measure, we excluded the 11 items that did not load above .40 on the first factor (see “Original 35-Item Pool for the SASSY” section for a breakdown of which items were excluded in this step). After these necessary scale adjustments had been made, an analysis of internal consistency was conducted with the remaining 24 items, yielding a Cronbach’s alpha of .80. However, analyses indicated that removing an additional four items would greatly increase internal consistency. This yielded a Cronbach’s alpha of .90 for the 20-item instrument. In the last step, the factor analysis was repeated, confirming a single-factor solution which explained 34% of the variance. Factor loadings for the items represented in the 20-item are shown in Table 2.

**Table 2:** Scale for the Assessment of Sexual Standards among Youth items and factor loadings across Studies 1 and 2

Item No.^a^	Study 1	Study 2 Wave 1	Study 2 Wave 2
1	.63	.41	.45
2	.40	.32^b^	.32^b^
3	.62	.50	.52
5	.51	.55	.52
6	.62	.58	.60
7	.46	.47	.54
8	.65	.63	.67
9	.71	.64	.68
10	.51	.62	.60
11	.56	.50	.53
12	.58	.57	.53
14	.53	.52	.56
18	.64	.59	.61
21	.54	.55	.53
24	.55	.60	.64
25	.45	.53	.54
26	.41	.46	.48
28	.53	.59	.63
33	.64	.58	.60
35	.43	.53	.60

^a^Item wording may be obtained from “Original 35-Item Pool for the SASSY” section. Item numbers match Appendix

^b^Item did not load sufficiently (cutoff >.40)

## Study 2

A subsequent study was conducted in two waves to examine whether the factor structure would replicate in a different sample using a slightly broader age group, and to examine the test–retest reliability, construct and convergent validity, and measurement invariance of instrument scores. Test–retest reliability was assessed by comparing scores across the two waves, which were eight weeks apart. Construct validity was addressed by assessing the relationship of participant scores on the new instrument to participant scores on the SDSS (Muehlenhard & Quackenbush, 1998). This scale was chosen because it is widely used for the assessment of sexual double standards (Bordini & Sperb, 2013). We expected there to be a strong positive relationship between the scale scores, because both scales have been designed to measure sexual double standard endorsement. However, we did not expect the relationship to near perfection, because the new instrument was additionally designed for a specific context, a specific target group, and to be more multifaceted compared to previous instruments. Lastly, convergent validity was established by assessing the relationship of participant scores on the new instrument to scores on gendered attitudes in another context, namely the family context. We expected to observe a positive weak to moderate relationship between the scale scores, because both scales have been designed to measure gendered attitudes.

## Method

### Participants

The original sample obtained at Wave 1 consisted of 873 adolescents and emerging adults. As in the first study, we excluded participants who indicated that they were attracted mainly or exclusively to members of their own sex, were attracted to both sexes equally, or were unsure of their sexual orientation (*n* = 55). The final sample used in the analyses consisted of 818 heterosexual adolescents and emerging adults at Wave 1 (this amounts to 93.7% of the original sample). In comparison with Wave 1, a further 202 participants were lost as they did not complete the Wave 2 questionnaire. This led to a final sample used in the analyses for Wave 2 of 616 heterosexual adolescents and emerging adults (this amounts to 70.6% of the original sample, and to 75.3% of the sample analyzed at Wave 1). A comparison between the participants who dropped out between Wave 1 and Wave 2 (*N* = 202) and participants who completed both waves (*N* = 616) showed no significant differences in gender, age or scores on the variable of interest (scores on the new instrument). Sample characteristics are shown in Table 3.

**Table 3:** Sample characteristics in Study 2

		Wave 1 (*N* = 818)				Wave 2 (*N* = 616)			
		Male (*n* = 340) (41.6%)		Female (*n* = 478) (58.4%)		Male (*n* = 246) (39.9%)		Female (*n* = 370) (60.1%)	
Age in years (*M*, SD)		20.76 (2.82)		20.81 (2.68)		20.80 (2.83)		20.79 (2.65)	
Ethnicity	Native Dutch	242 (80.4%)		323 (76.7%)		172 (79.6%)		258 (78.7%)	
	Of other ethnicity	59 (19.6%)		98 (23.3%)		44 (20.4%)		70 (21.3%)	
Education (*n*, %)	Lower	49 (15.4%)		53 (12.1%)		36 (15.9%)		37 (10.9%)	
	Intermediate	105 (33.0%)		129 (29.5%)		73 (32.2%)		100 (29.4%)	
	Higher	164 (51.6%)		255 (58.4%)		118 (52.0%)		203 (59.7%)	
Sexual experience (*n*, %)	No Yes	111 (34.9%) 207 (65.1%)		107 (23.7%) 344 (76.3%)		87 (37.3%) 146 (62.7%)		84 (24.0%) 266 (76.0%)	

Some variables contained missing cases. Valid score percentages are presented

### Procedure

This study was granted ethical approval by the Ethics Committee of the Faculty of Social and Behavioral Sciences at Utrecht University (Reference: FETC15-003). Data collection was outsourced to CentERdata, Institute for Data Collection and Research, which is attached to Tilburg University, the Netherlands, and was carried out using the LISS (Longitudinal Internet Studies for the Social Sciences) panel. The LISS panel is a representative sample of Dutch individuals who participate in monthly Internet surveys from the comfort of their own homes (in exchange for a small reward). The panel is based on a true probability sample of (approximately 5000) households drawn from the population register. Households without a computer and Internet access are provided these by LISS. A random selection of LISS panel members from those households was invited to participate in the study. The number of eligible candidates in each household varied, according to how many household members subscribe to the panel and whether they fit our age inclusion criterion. The specific sample included thus consisted of a unique draw from the participants in the LISS panel. More information about the panel can be found on their Web site (www.lissdata.nl). The method of data collection provided us with a sample of community adolescents and emerging adults aged between 16 and 25 years that was large enough for a solid validation process. The use of a panel made acquiring this sample more feasible as there was a known response rate within the panel and adequate oversampling could be provided. Moreover, using an Internet panel was of added value because our study involved rather personal questions and the Internet offers relative anonymity. Participants ticked a box stating that they understood that the questions would be of a sexual nature and that they wanted to continue to the questionnaire. The study was described to them as “a study on young people and sexuality.” They were further informed that they could cease their participation at any time. No parental consent was needed, because the minimum age for completing the questionnaire was 16. Data collection took place between May and July of 2014. To enable test–retest reliability to be examined, the same participants completed the questionnaire in two waves, the second wave taking place eight weeks after the first.

### Measures

The revised instrument described in Study 1, now consisting of 20 items, was administered to participants in both Wave 1 and Wave 2. Participants indicated their degree of agreement on a six-point scale ranging from “1 = completely disagree” to “6 = completely agree.” See “Original 35-Item Pool for the SASSY” section for English-language item wording.

#### Demographics

Gender, age, education level, and sexual experience were assessed in an identical manner to Study 1.

#### Construct Validity

The Sexual Double Standard Scale (Muehlenhard & Quackenbush, 1998) was included in the survey in order to examine construct validity. The scale contained 20 items on which participants could indicate their degree of agreement on a four-point scale ranging from “1 = completely disagree” to “4 = completely agree.” In our study, we obtained a Cronbach’s alpha of .52 for this scale. An example item is: “It is just as important for a man to be a virgin when he marries as it is for a woman.”

#### Convergent Validity

Scores on the constructs used to assess convergent validity were taken from the longitudinal database of the LISS panel. Complete data were available for 504 of the participants who had also completed the new instrument and SDSS questionnaires.

*Family gender norms* Questions were derived from the European Values Study (EVS) (2016). Higher scores on this measure indicated more conservative family gender norms. The scale contained seven items, rated on a five-point scale ranging from “1 = completely disagree” to “5 = completely agree.” The items formed a reliable scale, with a Cronbach’s alpha of .75 in this study. An example item is: “A child who is not yet attending school is likely to suffer if his or her mother has a job.”

*Traditional values* Questions were derived from the European Social Survey (ESS) (2016). Higher scores on this measure indicated more conservative gender norms for child-rearing. The scale contained four items, rated on a five-point scale ranging from “1 = completely disagree” to “5 = completely agree.” The items formed a reliable scale, with a Cronbach’s alpha of .72 in this study. An example item is: “Generally speaking, boys can be brought up more liberally than girls.”

### Analytical Strategy

First, the factor structure and internal consistency of the new instrument were reassessed. We employed a confirmatory factor analysis to this purpose. Subsequently, analyses were performed to ascertain test–retest reliability, construct validity, and convergent validity. Lastly, measurement (in)variance was examined across time, gender, age, education, sexual experience level, and ethnicity using confirmatory factor analysis. There were no missing values: therefore, no missing data handling procedure was needed.

## Results

### Factor Structure

The factor structure of the new instrument was reassessed using confirmatory factor analysis with principal axis factoring. The Kaiser–Meyer–Oklin value was .91 for both Wave 1 and Wave 2, which is above the recommended cutoff value of .60 (Kaiser, 1970, 1974), and Bartlett’s Test of Sphericity (Bartlett, 1954) was statistically significant in both Wave 1 and Wave 2, supporting factorability. The analysis showed that all items, except one, loaded above .40 and sufficiently strong on the first factor in both Wave 1 and Wave 2, supporting a one-factor solution. Item 2 (Girls like boys who take the lead in sex), however, loaded somewhat lower in both Wave 1 and Wave 2. Based on these factor loadings, we decided to drop Item 2. Subsequent analyses were, therefore, performed with the 19-item instrument. The single factor of the 19-item instrument explained 32% of the variance in Wave 1 and 34% of the variance in Wave 2. Factor loadings are shown in Table 2. This final set of items was named the Scale for the Assessment of Sexual Standards among Youth (SASSY). The final 19-item instrument can be found in “Original 35-Item Pool for the SASSY” section. Mean scores on the separate SASSY items as a function of gender are shown in Table 4.

**Table 4:** *t*-tests of separate SASSY item means as a function of gender (Study 2)

Item No.	Wave 1 (*N* = 818)		Effect size	Wave 2 (*N* = 616)		Effect size
	Male [*M* (SD)] (*n* = 340, 41.6%)	Female [*M* (SD)] (*n* = 478, 58.4%)	Cohen’s *d*	Male [*M* (SD)] (*n* = 246, 39.9%)	Female [*M* (SD)] (*n* = 370, 60.1%)	Cohen’s *d*
Overall mean	2.29 (.78)**	2.12 (.65)**	.24	2.28 (.78)**	2.09 (.67)**	.28
1	1.54 (1.03)***	1.23 (.67)***	.37	1.55 (1.02)***	1.29 (.74)***	.30
3	1.79 (1.02)	1.84 (1.02)	−.05	1.87 (1.02)	1.91 (1.02)	−.04
5	3.07 (1.51)***	2.50 (1.45)***	.39	2.81 (1.51)*	2.56 (1.38)*	.17
6	2.34 (1.35)*	2.14 (1.28)*	.15	2.39 (1.26)*	2.13 (1.27)*	.21
7	2.97 (1.58)	3.14 (1.54)	−.11	2.91 (1.55)	2.98 (1.53)	−.05
8	1.78 (1.09)*	1.62 (.96)*	.16	1.95 (1.18)*	1.72 (1.05)*	.21
9	1.83 (1.15)**	1.61 (1.00)**	.21	1.91 (1.13)**	1.66 (.95)**	.24
10	2.97 (1.37)	2.84 (1.36)	.10	2.95 (1.34)	2.82 (1.30)	.10
11	2.90 (1.51)**	2.63 (1.42)**	.18	2.85 (1.51)*	2.60 (1.42)*	.17
12	2.26 (1.27)***	1.71 (1.07)***	.48	2.21 (1.22)***	1.65 (.99)***	.51
14	1.79 (1.23)	1.82 (1.22)	−.02	1.80 (1.24)	1.71 (1.09)	.08
18	1.38 (.84)**	1.21 (.61)**	.22	1.45 (.94)**	1.25 (.65)**	.26
21	1.69 (1.07)	1.56 (1.00)	.13	1.69 (1.06)	1.61 (1.01)	.08
24	2.18 (1.42)*	1.97 (1.30)*	.16	2.15 (1.44)**	1.86 (1.18)**	.23
25	2.88 (1.52)	2.73 (1.52)	.10	2.97 (1.50)***	2.55 (1.41)***	.29
26	2.86 (1.26)	2.71 (1.15)	.13	2.88 (1.25)	2.69 (1.21)	.16
28	2.40 (1.38)	2.42 (1.41)	−.01	2.37 (1.43)	2.31 (1.30)	.04
33	1.83 (1.12)***	1.53 (.96)***	.29	1.79 (1.06)**	1.53 (.89)**	.27
35	3.04 (1.55)	3.09 (1.52)	−.03	2.93 (1.54)	2.80 (1.49)	.09

* *p* < .05; ** *p* < .01; *** *p* < .001

### Internal Consistency

The reliability of the 19-item SASSY scale was assessed in both Wave 1 and Wave 2. Cronbach’s alphas obtained were well above the cutoff point for a reliable scale: .89 in Wave 1 and .90 in Wave 2.

### Test–Retest Reliability

The correlation between the Wave 1 and Wave 2 SASSY data was substantial and highly significant (see Table 5), and within-gender scores on the SASSY did not differ significantly between Wave 1 and Wave 2.

**Table 5:** Correlations between SASSY and other measured constructs

	1.	2.	3.	4.	5.
1. SASSY Wave 1 (*n* = 818)	–				
2. SASSY Wave 2 (*n* = 616)	.70**	–			
3. SDSS Wave 1 (*n* = 818)	.53**		–		
4. Family gender roles (*n* = 504)	.21**	.23**	.24**	–	
5. Traditional values (*n* = 504)	.38**	.39**	.40**	.44**	–

Owing to the gendered nature of sexual double standards, results were additionally examined separately for gender. No differences in significance levels emerged, and there were only slight variations in correlation strength. Therefore, correlations aggregated over gender are shown

* *p* < .05; ** *p* < .01

### Construct Validity

The correlation between the SASSY and SDSS (Wave 1) was large (See Table 5).

### Convergent Validity

A small but significant, positive correlation was found between SASSY (in both Wave 1 and Wave 2) and Family Gender Norms (see Table 5), indicating that increased sexual double standard endorsement was related to less liberal family gender norms (toward women). A moderate significant positive correlation was found between SASSY (in both Wave 1 and Wave 2) and Traditional Values (see Table 5), indicating that increased sexual double standard endorsement was related to less liberal gender norms for child-rearing.

### Measurement Invariance

Lastly, measurement (in)variance was examined across time, gender, age, education, sexual experience level, and ethnicity using confirmatory factor analysis. We assessed configural invariance (requires that model fit is acceptable across groups), metric invariance (requires that factor loadings are invariant across groups), and scalar (or strong) invariance (requires that item intercepts are invariant across groups), as proposed by Steenkamp and Baumgartner (1998). As cited in Steenkamp and Baumgartner, measurement invariance refers to “whether or not, under different conditions of observing and studying phenomena, measurement operations yield measures of the same attribute.” In other words, whether a scale assesses true differences between groups or whether differences result from systematic biases. We used the standard Root Mean Square Error of Approximation (RMSEA) cutoff of <.05, with PCLOSE non-significant, and CFI > .90 to examine goodness of fit. To examine nested model differences, *χ* ^2^ difference tests were conducted. If this test was non-significant, measurement invariance is presumed to be present. However, we additionally used the decrease in CFI between the nested models, because the *χ* ^2^ difference test is sensitive to sample size, whereas CFI is more robust (Milfont & Fischer, 2015). If the *χ* ^2^ difference test was significant, but nested models differ by no more than .01 in CFI, measurement invariance is concluded to be present, regardless of the significant *χ* ^2^ difference test (Cheung & Rensvold, 2002).

The fit of the factor model was good: *χ* ^2^ (131) = 449.518, RMSEA = .055 (PCLOSE .077) and CFI = 0.932. All factor loadings were >.41. As shown in Table 6, the instrument showed configural and metric measurement invariance across gender, age, educational level, sexual experience level, and ethnicity, and configural, metric, and scalar measurement invariance across time.

**Table 6:** Tests of invariance constraints (1 configural, 2 metric and 3 scalar) for gender, age, education, sexual experience, time, and ethnicity

				*χ* ^2^ test of model fit			*χ* ^2^ difference test			Decision
Hierarchical invariance testing steps		CFI	RMSEA	Value	*df*	*p*	Δ *χ* ^2^	Δ *df*	*p*	
*Test of invariance constraints for gender (male vs. female)*										
1	Noninvariant	.91	.04	665.78	262	<.001				Invariance accepted
2	*λ* ^1^ invariant	.91	.04	700.95	281	<.001	35.16	19	.013	Invariance accepted
3	*λ* ^τ^ invariant	.89	<.05	823.96	300	<.001	123.01	19	<.001	Invariance rejected
*Test of invariance constraints for age (16*–*18, 19*–*21, 22*–*26)*										
1	Noninvariant	.90	.04	851.36	393	<.001				Invariance accepted
2	*λ* ^1^ invariant	.90	.04	906.09	431	<.001	54.72	38	.039	Invariance accepted
3	*λ* ^τ^ invariant	.89	.04	993.81	469	<.001	87.73	38	<.001	Invariance rejected
*Test of invariance constraints for education (low, moderate, high)*										
1	Noninvariant	.92	.03	739.07	393	<.001				Invariance accepted
2	*λ* ^1^ invariant	.91	.03	811.57	431	<.001	72.49	38	.001	Invariance accepted
3	*λ* ^τ^ invariant	.89	.04	916.84	469	<.001	105.27	38	<.001	Invariance rejected
*Test of invariance constraints for sexual experience (no vs. yes)*										
1	Noninvariant	.93	.04	586.67	262	<.001				Invariance accepted
2	*λ* ^1^ invariant	.93	.04	601.96	281	<.001	15.29	19	.704	Invariance accepted
3	*λ* ^τ^ invariant	.91	.04	673.56	300	<.001	71.60	19	<.001	Invariance rejected
*Test of invariance constraints between measurement waves (Wave 1 vs. Wave 2)*										
1	Noninvariant	.91	<.05	1073.56	262	<.001				Invariance accepted
2	*λ* ^1^ invariant	.91	<.05	1094.46	281	<.001	20.90	19	.342	Invariance accepted
3	*λ* ^τ^ invariant	.91	.04	1126.07	300	<.001	31.61	19	.035	Invariance accepted
*Test of invariance constraints for ethnicity (Native Dutch vs. Non*-*Native Dutch)*										
1	Noninvariant	.92	.04	614.85	262	<.001				Invariance accepted
2	*λ* ^1^ invariant	.92	.04	644.57	281	<.001	29.72	19	.055	Invariance accepted
3	*λ* ^τ^ invariant	.91	.04	705.75	300	<.001	61.18	19	<.001	Invariance rejected

## Discussion

As the concept of the sexual double standard has evolved, along with changes in the display of gendered behavior in dating and sexuality, and negative effects of sexual double standard endorsement on sexual health are evident (Bordini & Sperb, 2013; Crawford & Popp, 2003; Fugère et al., 2008; Sanchez et al., 2012), the development of modernized methods and measures is warranted. In response to this call, this study proposed a new, multifaceted, and one-dimensional 19-item scale to measure sexual double standard endorsement.

The SASSY demonstrated excellent one-factor model fit, internal consistency, test–retest reliability, convergent, and construct validity, and showed configural and metric measurement invariance across gender, age, education level, and sexual experience level and configural, metric, and scalar measurement invariance across time. Overall, this speaks for the use of the scale in future studies.

Of course, there were also some limitations to our study. First off, we note that no scalar measurement invariance was found across gender, age, education level, and sexual experience level. Strictly speaking, this would mean that (since both configural and metric invariance do hold) assessing structural relationships across variables using the SASSY is advisable, but comparing group means is not. However, measurement invariance is often ignored in (validation) studies altogether, and when it is not, strict forms of invariance (such as scalar invariance) only rarely hold (Van De Schoot, Schmidt, De Beuckelaer, Lek, & Zondervan-Zwijnenburg, 2015). Therefore, we do not see a reason to be overly cautious in comparing group means.

Secondly, although we were mindful of deleting entire themes through the deletion of items based on statistical arguments, for the theme of “gender violations” all items had to be dropped, based on either the factor loadings or the subsequent reliability analysis. However, since this was the only one of the established themes that dropped out completely in the process of creating the final instrument, we do not think that the multifaceted nature of the scale was compromised. We additionally assessed whether there were any commonalities to be detected among the deleted items (for instance, a lower degree of variability, compared to the retained items, that they shared), but this did not appear to be the case.

The present study used self-report data. As the instrument was designed to assess the individual’s attitude toward perceived social norms concerning sexuality for boys and girls, socially desirable responding can never be ruled out completely. We also note that using either a between- or within-subjects design in sexual double standard research may lead to different results, also regarding socially desirable responding. Both types of designs have previously been used in this field, generally assessing either individual double standards (mostly using within-subject designs) or perceptions of societal double standards (mostly using between-subject designs) (Crawford & Popp, 2003). Although it remains up for discussion whether the SASSY is more a measure of individual or societal double standards (or a combination), we believe it leans more toward the societal double standard. It could, therefore, be argued that it is an instrument that is most suitable for assessment in between-subject designs (Crawford & Popp, 2003). It is possible, that, as a result of the gender comparison inherently present in the scale, participants may be somewhat more inclined toward socially desirable responding and report a similar standard for boys and girls. Previous research shows that this is, however, not necessarily the case, even when assessing the SDS in a between-subjects design (Sakaluk & Milhausen, 2012).

In the future, the instrument might be adapted for use in sexual minority samples as well. However, as the sexual double standard is a highly heteronormative phenomenon, it seemed best to focus first on heterosexual populations.

## Appendix: Original 35-Item Pool for the SASSY

Below you will find a number of statements about boys and girls concerning sexuality. The statements refer to boys and girls, but they are also meant for young men and women. Please read the statements carefully and indicate whether or not you agree with each statement. We are only interested in your honest opinion; there are no right or wrong answers.

1. *Once a boy is sexually aroused, a girl cannot really refuse sex anymore.*
2. Girls like boys who take the lead in sex.^c^
3. *I think that a girl who takes the initiative in sex is pushy.*
4. Boys who jump into bed with anyone disgust me.^a^
5. *I think it is more appropriate for a boy than for a girl to date different people at the same time.*
6. *Girls should act in a more reserved way concerning sex than boys.*
7. *I think it is more appropriate for a boy than for a girl to have sex without love.*
8. *A boy should be more knowledgeable about sex than a girl.*
9. *I think sex is less important for girls than for boys.*
10. *I think it is normal for boys to take the dominant role in sex.*
11. *I think sexually explicit talk is more acceptable for a boy than for a girl.*
12. *Sometimes a boy should apply some pressure to a girl to get what he wants sexually.*
13. Girls who jump into bed with anyone disgust me.^a^
14. *It is more important for a girl to keep her virginity until marriage than it is for a boy.*
15. There are no longer any differences between men and women when it comes to sexuality.^a^
16. Romance is more important for girls than for boys.^a^
17. I think girls can have just as many one night stands as boys.^a^
18. *Boys are more entitled to sexual pleasure than girls.*
19. Boys like girls who take the lead in sex.^a^
20. I admire girls who behave in a masculine way.^a^
21. *It is not becoming for a girl to have unusual sexual desires.*
22. I admire a boy who is a virgin when he gets married.^a^
23. I admire boys who behave in a feminine way.^b^
24. *Sex is more important for boys than for girls.*
25. *It is more important for a girl to look attractive than it is for a boy.*
26. *Boys and girls want completely different things in sex.*
27. It’s best for girls not to have sex with too many different boys, otherwise people will think badly of them.^a^
28. *I think cheating is to be expected more from boys than from girls.*
29. I think it’s a good thing if boys let girls take the initiative for sex.^a^
30. Love is really more important than sex for boys.^a^
31. Sexually active girls create problems in relationships.^b^
32. I feel sorry for girls who are still virgins when they get married.^b^
33. *I think it is important for a boy to act as if he is sexually active, even if it is not true.*
34. Girls are more entitled to sexual pleasure than boys.^b^
35. *I think it is more appropriate for a boy than for a girl to masturbate frequently.*

*Note.* Italicized items are items that have been retained in the final version of the instrument. Original Dutch item wording can be obtained upon request.

^a^Indicates an item that was removed based on the factor analysis in Study 1.

^b^Indicates an item that was removed based on the subsequent reliability analysis in Study 1.

^c^Indicates an item that was removed based on the factor analysis in Study 2.

## References

[CR1] Allen L (2003). Girls want sex, boys want love: Resisting dominant discourses of (hetero) sexuality. Sexualities.

[CR2] Arnett JJ (2000). Emerging adulthood: A theory of development from the late teens through the twenties. American Psychologist.

[CR3] Bartlett MS (1954). A note on the multiplying factors for various *χ*^2^ approximations. Journal of the Royal Statistical Society Series B.

[CR4] Bay-Cheng LY (2015). The agency line: A neoliberal metric for appraising young women’s sexuality. Sex Roles.

[CR5] Bermúdez M, Castro A, Gude F, Buela-Casal G (2010). Relationship power in the couple and sexual double standard as predictors of the risk of sexually transmitted infections and HIV: Multicultural and gender differences. Current HIV Research.

[CR6] Bordini GS, Sperb TM (2013). Sexual double standard: A review of the literature between 2001 and 2010. Sexuality and Culture.

[CR7] Caron SL, Davis CM, Halteman WA, Stickle M (1993). Predictors of condom related behaviors among first year college students. Journal of Sex Research.

[CR8] Cheung GW, Rensvold RB (2002). Evaluating goodness-of-fit indexes for testing measurement invariance. Structural Equation Modeling.

[CR9] Collins WA, Welsh DP, Furman W (2009). Adolescent romantic relationships. Annual Review of Psychology.

[CR10] Crawford M, Popp D (2003). Sexual double standards: A review and methodological critique of two decades of research. Journal of Sex Research.

[CR11] Eaton AA, Rose S (2011). Has dating become more egalitarian? A 35 year review using sex roles. Sex Roles.

[CR12] Emmerink PMJ, van den Eijnden RJJM, Ter Bogt TFM, Vanwesenbeeck I (2016). Psychosexual correlates of sexual double standard endorsement in adolescent sexuality. Journal of Sex Research.

[CR13] European Social Survey. (2016). Retrieved from http://www.europeansocialsurvey.org/.

[CR14] European Values Study. (2016). Retrieved from http://www.europeanvaluesstudy.eu/.

[CR15] Fugère MA, Escoto C, Cousins AJ, Riggs ML, Haerich P (2008). Sexual attitudes and double standards: A literature review focusing on participant gender and ethnic background. Sexuality & Culture.

[CR16] Hayes RM, Lorenz K, Bell KA (2013). Victim blaming others rape myth acceptance and the just world belief. Feminist Criminology.

[CR17] Horne S, Zimmer-Gembeck MJ (2005). Female sexual subjectivity and well-being: Comparing late adolescents with different sexual experiences. Sexuality Research and Social Policy.

[CR18] Horne S, Zimmer-Gembeck MJ (2006). The Female Sexual Subjectivity Inventory: Development and validation of a multidimensional inventory for late adolescents and emerging adults. Psychology of Women Quarterly.

[CR19] Hyde JS (2005). The gender similarities hypothesis. American Psychologist.

[CR20] Kaiser HF (1970). A second generation little jiffy. Psychometrika.

[CR21] Kaiser HF (1974). An index of factorial simplicity. Psychometrika.

[CR22] Kehily M (2001). Bodies in school young men, embodiment, and heterosexual masculinities. Men and Masculinities.

[CR23] Kiefer AK, Sanchez DT (2007). Scripting sexual passivity: A gender role perspective. Personal Relationships.

[CR24] Kreager DA, Staff J (2009). The sexual double standard and adolescent peer acceptance. Social Psychology Quarterly.

[CR25] Marks MJ, Fraley RC (2005). The sexual double standard: Fact or fiction?. Sex Roles.

[CR26] Marks MJ, Fraley RC (2006). Confirmation bias and the sexual double standard. Sex Roles.

[CR27] Milfont TL, Fischer R (2015). Testing measurement invariance across groups: Applications in cross-cultural research. International Journal of Psychological Research.

[CR28] Moss-Racusin CA, Phelan JE, Rudman LA (2010). When men break the gender rules: Status incongruity and backlash against modest men. Psychology of Men & Masculinity.

[CR29] Muehlenhard CL, Quackenbush DM, Fisher TD, Davis CM, Yarber WL, Davis SL (1998). Sexual double standard scale. Handbook of sexuality-related measures.

[CR30] Petersen JL, Hyde JS (2010). A meta-analytic review of research on gender differences in sexuality, 1993–2007. Psychological Bulletin.

[CR31] Pleck JH, Sonenstein FL, Ku LC (1994). Attitudes toward male roles among adolescent males: A discriminant validity analysis. Sex Roles.

[CR32] Pulerwitz J, Barker G (2008). Measuring attitudes toward gender norms among young men in Brazil: Development and psychometric evaluation of the GEM scale. Men and Masculinities.

[CR33] Reidy DE, Shirk SD, Sloan CA, Zeichner A (2009). Men who aggress against women: Effects of feminine gender role violation on physical aggression in hypermasculine men. Psychology of Men & Masculinity.

[CR34] Reiss IL (1967). The social context of premarital sexual permissiveness.

[CR35] Sakaluk JK, Milhausen RR (2012). Factors influencing university students’ explicit and implicit sexual double standards. Journal of Sex Research.

[CR36] Sanchez DT, Crocker J, Boike KR (2005). Doing gender in the bedroom: Investing in gender norms and the sexual experience. Personality & Social Psychology Bulletin.

[CR37] Sanchez DT, Fetterolf JC, Rudman LA (2012). Eroticizing inequality in the united states: The consequences and determinants of traditional gender role adherence in intimate relationships. Journal of Sex Research.

[CR38] Shen AC, Chiu MY, Gao J (2012). Predictors of dating violence among Chinese adolescents: The role of gender-role beliefs and justification of violence. Journal of Interpersonal Violence.

[CR39] Sprecher S, Regan PC, McKinney K (1998). Beliefs about the outcomes of extramarital sexual relationships as a function of the gender of the “cheating spouse”. Sex Roles.

[CR40] Steenkamp JE, Baumgartner H (1998). Assessing measurement invariance in cross-national consumer research. Journal of Consumer Research.

[CR41] Szymanski DM, Henrichs-Beck C (2014). Exploring sexual minority women’s experiences of external and internalized heterosexism and sexism and their links to coping and distress. Sex Roles.

[CR42] Ter Bogt TFM, Engels RCME, Bogers S, Kloosterman M (2010). ”Shake it baby, shake it”: Media preferences, sexual attitudes and gender stereotypes among adolescents. Sex Roles.

[CR43] Tolman DL (2009). Dilemmas of desire: Teenage girls talk about sexuality.

[CR44] Tolman DL (2012). Female adolescents, sexual empowerment and desire: A missing discourse of gender inequity. Sex Roles.

[CR45] Van De Schoot R, Schmidt P, De Beuckelaer A, Lek K, Zondervan-Zwijnenburg M (2015). Editorial: Measurement invariance. Frontiers in Psychology.

[CR46] Vanwesenbeeck I (2009). Doing gender in sex and sex research. Archives of Sexual Behavior.

[CR47] Yasan A, Essizoglu A, Yildirim EA (2009). Predictor factors associated with premarital sexual behaviors among university students in an Islamic culture. International Journal of Sexual Health.

